# Niveau socioéconomique et processus du recours aux soins par les familles de patients souffrant de troubles psychiques au Burkina Faso

**DOI:** 10.11604/pamj.2014.17.207.3222

**Published:** 2014-03-16

**Authors:** Ahmed Yaogo, Alain Sommer, Pierre Moulaï, Saïd Chebili, Agnès Abaoub-Germain

**Affiliations:** 1 Etablissement Public de Santé Ville Evrard- 202 Avenue Jean Jaurès 93332 Neuilly sur Marne- France; 2 Conservatoire National des Arts et Métiers, Chaire d’économie et gestion des services de santé, 82 bld de sébastopol 75003 Paris, France

**Keywords:** Profession, catégorie sociale, niveau d'instruction scolaire, processus de recours aux soins, Profession, social category, level of schooling, health care seeking process

## Abstract

**Introduction:**

Le Burkina Faso a connu une amélioration constante depuis deux décennies de l'offre de soins en psychiatrie. De même, le taux d'alphabétisation sans cesse croissant s'accompagne d'une profonde modification des conceptions et des comportements. La présente étude visait à déterminer l′impact des déterminants socioéconomiques sur le processus du recours aux soins par les familles.

**Méthodes:**

Il s'est agi d'une enquête transversale portant sur 200 familles, menée dans le service de psychiatrie du Centre Hospitalier Universitaire Yalgado Ouédraogo de Ouagadougou. Variable à expliquer: premier recours aux soins par les familles (guérisseur traditionnel ou prières religieuses vs. consultations psychiatrique ou médicale). Variable explicative: catégorie socioprofessionnelle classée en suivant la nomenclature des professions et catégories socioprofessionnelles; niveau d’études. L'analyse statistique a été effectuée à l'aide du logiciel SAS version 9.2. Le test du Khi deux a été utilisé.

**Résultats:**

Il existait une association entre le choix du premier recours et la Profession et la catégorie socioprofessionnelledu « décideur » (p = 0.0006) ainsi que leniveau d’études du « décideur » (p = 0.0001).

**Conclusion:**

La Profession et Catégorie Sociale et le niveau d'instruction scolaire pourraient être un marqueur important dans les politiques visant à optimiser les processus de recours aux soins des patients dans le circuit de soins.

## Introduction

Le Burkina Faso connaît une utilisation encore faible des services de santé de façon générale [1]. L′exercice de la psychiatrie en particulier se heurte à de nombreux problèmes d′acceptabilité des soins par les malades et leurs familles [2]. Le système de santé au Burkina Faso est caractérisé par l'absence d'assurance maladie publique. L'assurance maladie proposée par des structures privées est inaccessible par la grande majorité de la population. En cas de maladie, c'est la solidarité au sein de la famille qui supplée à l'absence d'assurance maladie. Pour cela, les familles des malades mentaux utilisent un grand nombre de recours thérapeutiques autres que la médecine « moderne »; plus ou moins efficaces; engendrant de nombreux problèmes de planification. Le recours à la psychiatrie est souvent relégué en dernière position. De nombreuses mutations sont en cours au Burkina Faso tant sur le plan social que sur le plan de l'assistance psychiatrique. Sur le plan social, la solidarité jadis légendaire en Afrique est en passe d’être substituée par la culture de l'individualisme avec pour corollaire la nucléarisation de plus en plus prononcée de la famille. De plus, le taux d'alphabétisation sans cesse croissant s'accompagne d'une profonde modification des conceptions et des comportements. Quant à l'assistance psychiatrique, elle a connu une amélioration constante depuis deux décennies. Des efforts sont produits en vue d'améliorer l'offre de soins tant en qualité qu'en quantité. Quel est aujourd'hui l'impact de ces deux nouvelles donnes sur le processus de recours aux soins en cas de maladie mentale? Existe-t-il des paramètres socioéconomiques corrélés au processus du recours aux soins par les familles? Nous nous proposons d’étudier l′impact des déterminants socioéconomiques (niveau d′études, profession et catégorie sociale) sur le processus du recours aux soins par les familles. Notre hypothèse est que le processus de recours aux soins en cas de maladie mentale est associé au niveau d'instruction ou à la profession et catégorie sociale de la personne (membre de la famille) qui prend les décisions sur l'itinéraire thérapeutique des patients. Les résultats devront permettre de mieux organiser le processus de recours aux soins.

## Méthodes

**Objectif général:** Etudier l′impact des déterminants socioéconomiques (niveau d′études, profession et catégorie sociale) sur le processus du recours aux soins par les familles.

**Cadre d’étude:** Notre étude a été menée dans le service de psychiatrie du Centre Hospitalier Universitaire YalgadoOuédraogo(CHUYO)de Ouagadougou.

### Population d’étude

**Critères d'inclusion:** toutes les familles de patients qui présentaient une symptomatologie psychiatrique ayant motivé une consultation ou une hospitalisation dans le service de psychiatrie du CHUYO ont été contactées dans leurs chambres pour les uns et après les consultations pour les autres. Nous leur avons expliqués les objectifs et l'intérêt de l’étude. Après le consentement éclairé, nous avons retenu les patients qui avaient des accompagnants auprès de qui nous pouvions recueillir les informations nécessaires pour remplir les fiches préalablement établies. Pour les hospitalisés nous avons négocié avec la famille afin d'avoir un entretien en présence des personnes qui ont le plus pesé sur les choix des différents recours thérapeutiques. Si des informations nous manquaient, nous envisagions un deuxième contact afin de compléter ces informations. Pour les consultations externes, nous avons recueilli les informations auprès du patient et son accompagnant du jour. Si ce dernier disposait des informations sur le patient, nous l'incluions.

**Critères de non inclusion:** N’étaient pas inclus dans cette étude: les familles de patients qui ne consentaient pas à répondre aux différentes questions que nous administrions; les familles de patients auprès de qui des informations ne pouvaient être recueillies par défaut de personne ressource.

**Echantillonnage:** Nous avons effectué un échantillonnage par quota. Ce qui a permis de disposer de données sur les familles des patients hospitalisés et celles venues en consultation. Ainsi, on a pu disposer d'informations équilibrées suivant les différents degrés de gravité car ceci pourrait influencer l'itinéraire thérapeutique des patients

**Méthode de collecte des données :** Nous avons utilisé un questionnaire anonyme préalablement établi comme outil de collecte, validé par un pré-test. La technique de collecte utilisée a été l'entrevue. La collecte a été assurée par deux étudiants en fin d’études médicales.

**Considérations éthiques et déontologiques:** durant notre étude, nous avons pris des mesures en vue de minimiser le risque de causer des torts moraux ou émotifs. Nous nous sommes notamment abstenus de: violer la vie privée des répondants en posant des questions illicites; observer le comportement des répondants à leur insu; négliger de respecter certaines valeurs culturelles, traditions et tabous. Dans tous les cas, nous avons obtenu le consentement éclairé de tous les répondants avant l'entrevue et avons évité toute atteinte à la liberté individuelle.

**Type et période d’étude:** Il s'est agi d'une enquête transversale descriptive qui s'est déroulée durant les mois d'avril et mai 2008.

### Données

**Variable à expliquer:** Le premier recours aux soins par les familles (guérisseur traditionnel ou prières religieuses vs. consultations psychiatrique ou médicale).

**Variable explicative:** La catégorie socioprofessionnelle classée en suivant la nomenclature des professions et catégories socioprofessionnelles (PCS): cadres et professions intellectuelles supérieures; professions intermédiaires; employés; ouvriers. Pour des raisons d'effectifs et de similarité en termes de niveau de revenus et de conditions de vie nous avons regroupé certaines catégories en une seule catégorie [3].

**Analyse statistique:** L'objectif de cette étude a été de tester l'hypothèse suivante: la situation sociale, mesurée par la Profession et Catégorie Sociale des personnes ou le niveau d’études scolaires, est un facteur associé au processus du recours aux soins par les familles.

L'analyse statistique a été effectuée à l'aide du logiciel SAS version 9.2 et a comporté plusieurs étapes: premièrement, nous avons décrit les 200 sujets qui ont été inclus dans notre étude. Le test t de Student a été utilisé pour les variables quantitatives et le test du Chi2 pour les variables qualitatives.

## Résultats

Le Tableau 1 montre les processus de recours aux soins des familles des sujets inclus dans notre étude. Nous remarquons que les familles utilisent dans 56% des cas le guérisseur traditionnel ou les prières religieuses comme premiers recours. Le recours à la médecine moderne venant après l’échec de ce premier recours. Le Tableau 2 montre les résultats de l'analyse du lien entre la Profession et Catégorie Sociale du « décideur » ou le niveau d'instruction et le choix du premier recours. Nous avons observé une association statistiquement significative entre la Profession et Catégorie Sociale du « décideur » et le choix du premier recours (p = 0.0001). Nous avons par ailleurs observé une association statistiquement significative entre le niveau d’étude du « décideur » et le choix du premier recours (p = 0.0006).

**Tableau 1:** Répartition des patients en fonction du type de recours utilisé aux différentes étapes de leur itinéraire thérapeutique

	Recours thérapeutique utilisé				
	Guérisseur traditionnel	Prières religieuses	Consultation psychiatrique	Consultation médicale	
	%	%	%	%	p
Rang du recours					
Premier recours(n = 200)	32.0	24.0	21.0	23.0	0.0001
Deuxième recours (n = 136)	33.8	16.2	33.8	16.2	
Troisième recours (n = 72)	8.3	30.6	38.9	22.2	
Quatrième recours (n = 30)	0.0	26.7	66.7	6.7	
Cinquième recours (n = 2)	0.0	100.0	0.0	0.0	

**Tableau 2:** Répartition des « décideurs » au premiers recours en fonction de leur catégorie socioprofessionnelle et du niveau d'instruction

	Premier recours thérapeutique utilisé		
	Guérisseur traditionnel ou Prières religieuses	Consultation psychiatrique ou Consultation médicale	
	%	%	p
Profession et catégorie sociale			
Employés ou ouvriers	71.2	28.8	0.0001
(n = 118) (%)	34.1	65.9	
Cadres ou professions intermédiaires (n = 82) (%)			
Niveau d'instruction			
Aucun (n = 58) (%)	44.8	55.2	0.0006
Primaire (n = 74) (%)	81.1	18.9	
Secondaire (n = 36) (%)	33.3	66.7	
Supérieur (n = 32) (%)	43.8	56.3	

## Discussion

Le résultat principal de notre étude est que le processus de recours aux soins en cas de maladie mentale est associé au niveau d'instruction ou à la profession et catégorie sociale de la personne (membre de la famille) qui prend les décisions sur l'itinéraire thérapeutique des patients.

**Limites et mérites de l’étude:** l'interprétation de nos résultats devrait tenir compte d'un certain nombre de limites liées à l’étude:

**Type d’étude:** Nous avons mené une enquête transversale descriptive. Ce qui pourrait fournir des informations utiles sur les processus de recours aux soins de nos patients. Cependant, ce type d’étude nous a permis de faire le point sur cette question juste sur la période d’étude mais ne nous permet pas de dégager une tendance dans le temps.

**Collecte des données:** nous avons utilisé un questionnaire préalablement établi pour la collecte des données et les entrevues comme technique de collecte. Cette technique a eu pour avantages de convenir aux analphabètes; elle nous a permis aussi d’éclaircir les questions et d'augmenter le taux des réponses. Cependant, la présence de l'enquêteur a pu influer sur les réponses. De plus, le fait que les événements étaient rapportés à posteriori les a probablement rendus moins complets du fait de biais de remémoration. Néanmoins, le pré-test, le caractère anonyme du questionnaire constituent des éléments considérables en faveur de la fiabilité de nos données.

### Processus du recours aux soins

Dans notre série 21% des patients étaient venus au service de psychiatrie du CHUYO sans recours antérieurs. Le recours traditionnel et les prières religieusesétaientutilisés en première intention par 56% des patients. Le recours à la médecine moderne (44%) était assez peu utilisé face à une maladie mentale en première intention. Néanmoins, ce pourcentage ne répond pas à l'idée qui est qu'en Afrique, presque tous les malades mentaux passent par le guérisseur traditionnel avant d'entrer à l'hôpital. Déjà en 1988, Siranyan S. [4] faisait le constat que 48% des malades inauguraient l'itinéraire thérapeutique par l'hospitalisation. Nos résultats sont également similaires à ceux de Yaméogo D. [5] qui enregistrait 44,5% de patients qui avaient eu recours aux thérapies modernes en première intention. Ils sont par contre contraires à ceux de Sylla A. [6] qui trouvait en 2001 que les familles, pour « réparer » la déviance, faisaient souvent des recours aux soins traditionnels dans une grande discrétion, avant de s'adresser à l'hôpital. On assiste alors à une sorte de psycho palabre entre les différents membres de la famille aboutissant à la définition de l'itinéraire thérapeutique.

Dans l'ensemble, le niveau d'adoption de la consultation psychiatrique demeure insatisfaisant. Ainsi lorsque le malade présente une symptomatologie assez clémente, les familles font le choix d'une consultation chez le guérisseur traditionnel ou le thérapeute religieux espérant une cure radicale.

Dans notre étude, les prières religieuses étaient assez utilisées par les patients en première intention. Ceci pourrait s'expliquer par le fait qu'en Afrique, les institutions religieuses sont souvent couvertes de vertus miraculeuses. Les nombreuses tentatives de traitement associées à cet aspect miraculeux font naître une certaine curiosité qui conduit les parents des malades à aller « essayer voir ». Cette utilisation est une tradition ancienne. En Europe, ce phénomène a été observé avec le déclin des institutions romaines au moyen âge et la montée des croyances locales christianisées, la folie a dû s'accommoder et faire son entrée dans l’église [7].

### Caractéristiques sociodémographiques des « décideurs » du recours aux soins

#### Niveau d'instruction et processus de recours aux soins

La majorité des « décideurs » a un niveau d'instruction moyen dans notre série. Ce constat pourrait s'expliquer par le fait que nos « décideurs » issus de la ville capitale (Ouagadougou) en majorité ont plus de chances de scolarisation dans notre contexte. Par ailleurs, on pourrait penser que ce sont les « décideurs » instruits qui ont le plus le réflexe de consulter en milieu hospitalier en cas de maladie. Cette explication avait été utilisée par Siranyan S. [4] qui notait en 1988 que dans certains milieux ruraux au Burkina Faso, on pense que l'accès à l'hôpital est réservé à des personnes qui ont un certain niveau d'instruction. Ceci pourrait s'expliquer par le fait que les « décideurs » ayant un niveau d'instruction élevé seraient plus aptes à trouver des explications socio environnementales ou biomédicales à la souffrance psychique. Notre constat est similaire à celui de Yaméogo D [5]. Il constatait en effet que les patients qui faisaient le plus recours aux thérapies modernes étaient ceux qui avaient un niveau d'instruction satisfaisant (52,8% de niveau secondaire et 18,90% de niveau supérieur).

#### Profession et Catégorie Sociale et processus de recours aux soins

L'explication de l'association entre la Profession et Catégorie Sociale et le processus de recours aux soins est très complexe. Une des hypothèses possibles est celle de la « causalité sociale »[8]. Cette hypothèse implique que la situation sociale influe directement sur le processus de recours aux soins. En effet, la situation sociale est associée au stress vécu dans la vie quotidienne et à la réaction face au stress. Les événements de la vie peuvent être définis comme des expériences cognitives et émotionnelles qui perturbent le cours de la vie et demandent à la personne un réajustement de ses habitudes, de son comportement et de ses représentations. Cette capacité de réajustement est fonction du niveau de compréhension du stress vécu. Les plus favorisés disposent probablement de ressources cognitives et culturelles leur permettant de mieux repérer un état de stress vécu mais aussi à mieux y faire face par l'adéquation d'une demande [9]; ce qui leur permet en cas de difficultés de bénéficier de conseils avisés et ainsi de faire les meilleurs choix de recours thérapeutique, simplifiant ainsi le processus de recours aux soins. Nos résultats pourraient trouver une explication également dans la différence d'accès aux soins [9]; les catégories socioprofessionnelles supérieures pouvant être favorisées de par le niveau d'information, et de par l'accessibilité financière. En effet, face à la maladie, les catégories socioprofessionnelles supérieures peuvent se permettre de faire un choix de recours thérapeutique et avoir les moyens financiers pour y faire face; ce n'est pas le cas des catégories socioprofessionnelles défavorisées qui sont obligées d'ajuster leur choix à leur capacité de paiement.

## Conclusion

Notre étude montre que la situation sociale, mesurée par la Profession et Catégorie Sociale et le niveau d’études, est un facteur associé au processus de recours aux soins en cas de maladie mentale. La Profession et Catégorie Sociale et le niveau d'instruction pourraient donc être un marqueur important dans les politiques visant à optimiser les processus de recours aux soins des patients dans le circuit de soins. Les professionnels de santé devraient être particulièrement attentifs dans l'orientation des personnes ayant une situation sociale défavorisée et, envisager des conseils sur les parcours de soins. D'autres études devraient permettre de clarifier davantage cette problématique.
